# Influence of maternal oral microbiome on newborn oral microbiome in healthy pregnancies

**DOI:** 10.1186/s13052-023-01520-w

**Published:** 2023-10-15

**Authors:** Monica Russo, Maria Grazia Calevo, Gloria D’Alessandro, Matteo Tantari, Marco Migliorati, Ilaria Piccardo, Paola Polo Perucchin, Cesare Arioni

**Affiliations:** 1 Operative Unit of Neonatology, IRCCS Ospedale Policlinico San Martino, Largo Rosanna Benzi 10, Genoa, 16132 Italy; 2 Epidemiology and Biostatistics Unit, Scientific Direction, IRCCS Istituto Giannina Gaslini, Genoa, Italy; 3 Academy Unit of Obstetrics and Gynecology, IRCCS Ospedale Policlinico San Martino, Genoa, Italy; 4 Department of Surgical Sciences and Integrated Diagnostics (DISC). Orthodontics Department, Genoa University, Genoa, Italy

**Keywords:** Oral microbiome, Full-term newborn, Mother-infant colonization

## Abstract

**Background:**

Periodontal disease and its bacteria can be responsible for pregnancy complications and transmission of periodontal bacteria from mother to newborn.

**Methods:**

A salivary swab to 60 healthy, full-term newborns and their mothers was taken immediately after birth. The test was performed with Real Time PCR method to evaluate the expression of the gene through DNA amplification. The species considered were: Aggregatibacter actinomycetemcomitans, Porphyromonas gingivalis, Prevotella intermedia and Fusobacterium nucleatum ssp.

**Results:**

The newborn oral microbiome was composed primarily by saprophytes (98.38 + 4.88%), just like the mothers (98.8 + 3.69%). There was a statistically significant difference of the total microbiological density in newborns and mothers (p = 0.0001). Maternal and neonatal oral microbiome had a correlated total microbiological density only in 33.3% (N = 20/60) of cases. The analysis of the oral microbiome showed a pathological composition only in 12/60 babies (20%). The most frequent detected specie in newborns was Fusobacterium nucleatum (9/12 babies, 75%), as well as for the mothers (53.3%). However, the pathogen was present both in baby and his mother only in 3 dyads. Porphyromonas gingivalis showed the highest association mother-baby (4/12 dyads, 33%). Porphyromonas gingivalis was the pathogen with the highest bacterial load in the 12 mothers. We found a statistically significant difference in the total load of Porphyromonas gingivalis in mothers and babies (p = 0.02).

**Conclusions:**

There was a statistically significant difference in the richness of the microbiome from newborns and mothers. Even comparing the microbiological density in the oral cavity of the individual mother-child pairs, we did not find a significant concordance. These results seem to suggest a low influence of maternal oral microbiome on the richness of the oral neonatal one. We didn’t find mother-child concordance (p = 0.0001) in the presence of pathogenic periodontal micro-organisms. Fusobacterium nucleatum was the most frequent specie detected. Porphyromonas gingivalis instead was the bacteria with the higher possibility of transmission. In conclusion in our study maternal oral health doesn’t affect healthy, full-term newborns’ oral microbiome. Further studies are needed to understand the maternal influence on newborn’s oral microbiome and its effects on babies long-term health.

## Background

Even if the origin of the neonatal oral microbiome is poorly understood, it is clearly that richness and change in composition of neonatal oral microbiome impact both short and long term outcomes [1, 2]. It is assumed that the initial development and maturation of the neonatal oral microbiome is largely determinate by maternal-offspring exchanges of microbiome during and after delivery [3, 4]. There are evidences that newborn’s oral microbiome is influenced first of all by the mode of delivery [5, 6] and several studies demostred the impact of this on the infant’s future health. It has been infact demostred the influence of C-section in microbioma’s composition and the association with an increased incidence of celiac disease, asthma, type 1 diabetes and obesity [7–10]. Also use of use of antibiotics in pregnancy may increase offspring risk of childhood desease [11, 12]. However, accumulating evidences show that neonatal oral microbiome has a prenatal origin [13, 14]. Gingivitis is the most common oral disease in pregnancy. The species characterized by high pathogenicity are: Aggregatibacter actinomycetemcomitans, Phorphyromonas gingivalis, Tanarella forsythia and Treponema denticle. They are also added, with less pathogenicity, species such as the Prevotella intermedia and the Fusobacterium nucleatum. The latter is less harmful individually than the first, but a synergy between these two complexes can lead to an increase in virulence. The scientific literature, in the last twenty years, has a growing interest in showing that periodontal disease and its bacteria can be responsible for pregnancy complications such as preterm births or low birth weight and transmission of periodontal bacteria from mother to newborn [15]. Two pathogenic mechanisms have been proposed to explain the connection from maternal oral dysbiosis and placental and fetal biology: the direct pathway is based on the presence of gram-negative anaerobic bacteremia originating in the gingival biofilm [16], whereas the indirect pathway involves the production of pro-inflammatory markers which enter the bloodstream from the gingival submucosa [17].

The influence of maternal oral microbiome to early oral newborn colonization isn’t well understood and there is a need for more in-depth research to understand if cariogenic bacteria colonize the fetus’ mouth in healthy pregnancies too.

Oral diseases are progressive, cumulative and grow in complexity over time, which is why it is very important for good oral health to be established early in life.

In order to motivate the oral hygiene of women of childbearing age and to prevent future diseases in the systemic and oral health of children, we analized the neonatal oral microbiome to estabilsh if there is an influence of the maternal oral microbiome in healthy pregnancies on the neonatal mouth colonization at birth.

## Patients and methods

This Retropective observational cohort study was performed in the Neonatal Unit of the IRCCS Ospedale Policlinico San Martino Genoa, Italy in collaboration with the Academy Unit of Obstetrics and Gynecology and the Orthodontics Department of Genoa University between 2017 and 2018.

A total of 60 newborns (34 F, 26 M) and their mothers were enrolled to study their oral microbiome. The average birth weight was 3205.9 g, ranged 2600–3850 g. Women ages was ranging from 20 to 42 years old (mean 32.08 y.o.).

Inclusion criteria were: newborns must have to be born by vaginal delivery, a term (37th − 41th gestational week), in health and adeguate for gestional age (AGA). Babies just after birth must stay skin to skin with their mothers. Mothers must not have received antibiotics during delivery and must not have taken antibiotics during the last 6 mounths of pregnancy. Furthermore, the women in this study must not have drunk or eaten or brushed their teeth half an hour before the test. None of the mothers had received dental treatment during pregnancy.

Exclusion criteria were: newborns by caesarean section, prematurity (< 37th gestational week), newborns with difficult birth or any health problem who don’t permitte them to stay immediatly after birth skin to skin with the mother because they necessity hospitalization in Neonatal Intensive Care Unit.

The outcome of this study was to analize oral neonatal microbiome and to evaluate the trasmission in healthy pregnancies of the major pathogenic bacteries in oral diseases comparing it with the maternal oral cavity microbiome.

The study provides for the execution of a salivary swab to the neonate and his mother; it was a multi-site microbiological test. The samples were taken from the baby’s immediately after birth, before start breastfeeding, during skin to skin to accurately reflect colonization prior to and during birth. Samplings were obtained using steril paper cones swiped on the oral mucose of the babies and inserted into the periodontal sulcus of the mothers for at least 1 min. The test was performed with Real Time PCR method, to evaluate the expression of the gene through DNA amplification.

The microbial density in the test was expressed in number of bacteries (copies per µl).

The species considered for this research were: Aggregatibacter actinomycetemcomitans, Porphyromonas gingivalis, Prevotella intermedia and Fusobacterium nucleatum ssp.

Written informed consent was obtained from parents or caregiver of patients after full explanation of the study, in according to the declaration of Helsinki.

Data are described as mean and standard deviation (SD) or median and range for continuous variables, and as absolute and relative frequencies for categorical variables. Non-parametric analysis (Mann-Whitney U-test, Wilcoxon test) for continuous variables and the Chi square or Fisher’s exact test for categorical variables were used to measure differences between groups. P values ≤ 0.05 were considered statistically significant, and all P values were based on two-tailed tests. Statistical analysis was performed using SPSS for Windows (SPSS Inc, Chicago, Illinois USA).

Ethical clearance for the publication of this study was obtained from the Regional Ethical Commitee (CER Liguria: 415/2022 – DB id 12,565).

## Results

This study analyzed 60 babies’oral microbiome at birth. All infants were born at full term by vaginal, eutocic delivery. Newborns were adeguate for gestational age (AGA) and had not health problems.

We analyzed and compared newborn’s oral microbiome at birth with their mothers’oral microbiome to understand if it could be influenced by their mothers during pregnancy.

We found a microbial community density lower than 10^3^ copies per µl in 10/60 newborns (16.7%); 17/60 babies (28.3%) were between 10^3^ and 10^4^ copies per µl; 14/60 babies (23.3%) had microbiological density higher than 10^4^ but lower than 10^5^ copies per µl. Richer microbiome (> 10^5^ copies per µl) was found in 19/60 babies (31.7%), only 4 of them had a microbial community density > 10^6^ copies per µl.

There was a statistically significant difference of the total microbiological density in newborns and mothers (p = 0.0001). Maternal and neonatal oral microbiome had a correlated total microbiological density only in 33.3% (N = 20/60) of cases. 18 mothers and babies had both a microbiome > 10^5^ copies per µl. In 2 cases both mother and neonate had microbilogical density < 10^5^ copies per µl. In all the other couples, mothers had a richer microbiome > 10^5^ copies per µl, but their children weren’t.

The oral cavity microbiome of the 60 full term babies was composed primarily by saprophytes (98.38 ± 4.88%), just like the mothers (98.8 ± 3.69%). The analysis of the oral microbiome showed a pathological composition only in 12/60 babies (20%). Only 4/12 of these newborns had a richer microbiological density too (> 10^5^ copies per µl). In comparison, the oral microbiome of mothers showed the presence of pathogenetic bacteria in oral diseases in 80% (N = 48/60), so in 36/60 cases we didn’t find mother-child concordance (p = 0.0001) in the presence of pathogenic periodontal micro-organisms.

We studied the neonatal oral microbiome in these 12 babies and compared it with their mother’s.

Because mothers had richer microbiome than newborns, in these 12 dyads was confirmed a statistically difference in the total microbiological density (p = 0.002). In addition, comparing the percentage of saprofite and pathogens in the microbiome, there was also a significative difference in the microbiome composition of the 12 babies and their mothers (p = 0.03).

About investigation of the periodontal micro-organisms in the 12 babies, in 4/12 babies (33.3%) there was a polimicrobial contamination: in 1 case of them we found contamination by 3 different species, the other 3 cases was contaminated by 2 different species, they had higher total microbial community > 10^5^ copies per µl.

We found Fusobacterium nucleatum in 9/12 babies (75%): it was the most frequent specie present in these 12 newborns. Even in mothers Fusobacterium nucleatum was the most frequent oral bacteria detected: the frequence in the 60 mothers was high 53.3%. In 6 mothers of these 9 babies, Fusobacterium nucleatum wasn’t found, so only in three of the dyads the presence of Fusobacterium nucleatum spp was associated.

The frequence of the species Prevotella intermedia and Porphyromonas gingivalis in the babies’ oral microbiome was the same (N = 4/12; 33%), lower than Fusobacterium nucleatum. The frequence of Prevotella intermedia in all 60 mothers was 31.7% (N = 19/60). Prevotella intermedia was present in 4/12 mothers, but only in 2 of the 12 dyads (16.6%) Prevotella intermedia was observed in both mother and newborn. Porphyromonas gingivalis was present in 29/60 mothers (48.3%) and in 7 of the 12 mothers (58.3%). It showed an higher association mother-baby than all the others specie examinated because we observed trasmission of Porphyromonas gingivalis in 4 of the 12 dyads (33%).

Despite the specie with the highest bacterial load in newborns was Fusobacterium nucleatum (1266 ± 2634 copies per µl), Porphyromonas gingivalis was the oral pathogen with the highest bacterial load in the 12 mothers. There was a statistically difference in the total load of Porphyromonas gingivalis in mothers and babies (6459 ± 13,106 vs. 40 ± 60 copies per µl; p = 0.02). This difference wasn’t observed for the other species examinated, in fact the bacterial count of Fusobacterium nucleatum, Prevotella intermedia was similar in the 12 dyads mother-newborn (p > 0.05).

None of the 12 babies showed the presence of Aggregatibacter actinomycetemcomitans in their oral microbiome. Aggregatibacter actinomycetemcomitans was the less frequent in the mothers’ oral microbiome too: it was present in only 1 mother of the 12 dyads and in 7/60 mothers (11.7%).

## Discussion

Several studies confirm that the microbiome in the first years of life is fundamental for health in future. Even if the most of oral microbial colonization is traditionally considered to take place after birth, the beginning of the development of the microbiome seems to takes place just at the fetal level with the passage of bacteria through the placenta and then becomes more complex from birth onward [18]. The human gut is the first most rich microbiome with a great diversied microbial community, meanwhile the second is the oral cavity [19]. Many studies have shown the presence of a rich microbiome just in intestinal fetus and newborn: it is very likely that this is due to the fact that the fetus ingests large amounts of amniotic fluid during the last trimester of pregnancy. Several mechanisms have been proposed to explain how bacteria may be able to colonize the uterine cavity during pregnancy. In relation to healthy pregnancies, two main pathways are currently considered: by blood, through the placenta after translocation from the digestive tract (oral cavity and intestines) or by vertical trasmission from the vaginal tract. A recent systematic review summarize studies on oral microbiome in infants, children and adolescents: evidence indicate that a stable core microbiome is present just in newborns and it becomes more differentiated within the first four years of life [20].

In our study, the microbiological test executed at full term birth by a salivary swab, shows us the density of microbiological population in the mouth of the neonates and their mothers. We examinated the richness of oral microbial community of the babies immediately after birth, before skin to skin contact with the mother and before start breastfeeding, to reduce the influence of them on the density of the microbial community in the mouth at birth, so to accurately reflect colonization prior to and during birth. It is discussed if the delivery mode influence oral microbiome, some recent studies conclude that newborn microflora isn’t related to the kind of delivery but instead primarily driven by body habitats [1, 21]. In any case, to riduce the influence of delivery mode on microbial density, the babies of the study were born exclusively by vaginal delivery. Furthermore mothers were not given antibiotics during delivery and hadn’t taken antibiotics during the last 6 mounths of pregnancy. In spite of everything samples from the oral cavity of our neonates showed low microbial richness, infact only 4 babies had a very rich microbiome with more than 10^6^ copies per µl. There was a statistically significant difference in the richness of the microbiome from the 60 newborns and mothers (p = 0.0001). Even comparing the microbiological density in the oral cavity of the individual mother-child pairs, we did not find a significant concordance (33.3%). It is supposed that the presence of caries in mothers don’t influence microbiome density and composition in infants in the first year of life [22, 23], results of this study seem to suggest a low influence of maternal oral microbiome on the richness of oral newborns microbiome at birth too. A so low microbiological density in the mouth of the full term babies immediately after birth could also effort the consideration that, even if the the foetal microbiome colonization may begin already in utero, oral microbiome composition matures throughout the first period of life and it is shaped by factors including host genetics and the enviroment [14]. Microbiome is a dynamic ecosystem and most of the development of the composition and function of the child’s oral microbiome occurs in the first years [20] and in particular probably just in the first hours of life [3, 4, 24]. So, the contact with the mother and our interventions on the environment around the mother and the newborn just after birth are very important for future richness and composition of the infant oral microbiome: the initial microbial exchanges between mother and infant at birth are fundamental as these early colonisers play a very important role in the development of the neonate’s immune system and long term in the activity and function of the microbiome [25].

Oral microbiome is a complex community and its composition and persistence is strictly influenced by food, oral hygiene practices and salivary flow [26]. Several studies investigate oral microbiome composition using advanced techniques of DNA sequencing, they found that the main phyla present in oral cavity are: Firmicutes, Bacteroides, Proteobacteria, Actinobacteria, Fusobacteria and Spirochaetes [20, 24, 27, 28]. To show the influence of maternal oral pathogens on the babies, we analized the presence at full term birth of major pathogenetic bacteries in oral diseases: Aggregatibacter actinomycetemcomitans, Porphyromonas gingivalis, Prevotella intermedia and Fusobacterium nucleatum ssp.

Oral diseases are progressive, cumulative and grow in complexity over time, which is why it is very important for good oral health to be established early in life. The etiology of both dental caries and periodontal disease is polymicrobial and occurs when there is a shift in the overall ecological balance of microbes in the oral cavity [29].

It is known that the oral health and oral microbiome of a woman may directly affect her pregnancy and her developing fetus: if the mother has periodontal disease, she has higher risk for giving preterm birth, for delivering a low-birth-weight infant, for preeclampsia, and 3.4 times higher risk for preterm birth plus delivery of an infant of low birth weight [15]. So, even if there is still insufficient evidence to conclude, it has been proposed that periodontal pathogens or their products some way reach the placenta and spread beyond it to the fetus [30].

Studies have found that in the case of oral diseases (gingivitis or periodontitis), bacteria in the oral cavity may reach amniotic fluid through transient bacteremia, indicating that maternal microbes may be transmitted to the amniotic fluid with blood.

The available studies have focused their attention on the immediate complications of fetal and neonatal colonization by these pathogenic germs of the oral cavity, but it isn’t yet understood whether transmission of these pathogens is possible even in uncomplicated pregnancies, in healthy full-term infants and it is not possible to exclude that a colonization already at birth can determine a worse prognosis of oral health in the child.

The genera considered in this study, Aggregatibacter actinomycetemcomitans, Porphyromonas gingivalis, Prevotella intermedia, Fusobacterium nucleatum, are all Gram negative oral pathogens responsible for periodontal disease. They are characterized by the production of various metabolites and virulence factors that lead to the destruction of periodontal tissue or inactivation of the local defenses of the immune system.

We found that the newborns’ oral cavity microbiome was composed primarily by saprophytes. The analysis of the oral microbiome showed in fact a pathological composition only in 20% of babies, this data doesn’t correlate with the incidence of periodontal pathogens in the mothers (N = 48/60; 80%). So in our samples there weren’t significative trasmission of periodontal micro-organisms and we could conclude that the oral health and oral microbiome of the mothers don’t affect a full term pregnancy. So the hypothesis of colonization in utero is still controversial and these datas would make us suspect that in some pregnancies, but not in all, there may be a bacteremia which may be followed by a complication of gestation. However, from the analysis of these data it would not be clear what is the mechanism that leads to the unleashing of bacteremia, since the presence of pathogens of the oral cavity in our 12 children did not correlate with the highest maternal bacterial charges.

Fusobacterium nucleatum was the most frequent specie detected in both mothers and babies, although again there was no correlation in the dyads. Fusobacterium nucleatum is an opportunistic periodontal pathogen of the oral cavity, but the dysbiosis can determine its pathogenicity with local and systemic consequences. We can’t determinate what influence will have for the future the presence of Fusobacterium nucleatum in the oral cavity immediately at birth but it is interesting that, even if in few babies of our sample, this bacteria was in some way able to trough the placenta also in healthy gestations.

Analysis of the other pathogenic genera of the oral cavity of our study showed a lower incidence of presence than Fusobacterium nucleatum in the oral mouth at birth. Porphyromonas gingivalis showed an higher correlation mother-babies (N = 4/12; 33%) than all the other genera examinated, so it could be the bacteria with the higher possibility of trasmission, but it is at the moment a too low evidence to demostrate it. None of the babies showed the presence of Aggregatibacter actinomycetemcomitans in their oral microbioma. Even in mothers it was the bacterium with the lowest incidence in the oral cavity.

In 4 babies there was a polimicrobial contamination too. Sometimes the association between two or more pathogens can be even more aggressive and harmful, polymicrobial infections in fact can be more serious than those caused by a single pathogen, when the interactions between individual species turn a mixed infection into a synergistic infection.

We cannot still know exactly what influence the presence of Fusobacterium nucleatum and other periodontal microorganisms in the oral cavity just at birth will have for the future infant oral health, but we could assume for these newborns a greater future risk of pathology if the balance between the different species of the microbiome under the influence of environmental factors will be broken.

**In conclusion**, in our study maternal oral microbiome doesn’t influence healthy, full-term newborn’s oral microbiome. There are currently not enough studies that analyze the oral microbiome at full-term birth and compare it with the maternal one. Research into oral pathogenic microorganisms in newborns has also focused on the effects on gestation, but the possible transmission to healthy full-term infants and its possible effects on long-term health has not yet been investigated. Further studies are needed.

## Data Availability

The datasets generated during the current study are available from the corrisponding author on reasonable request.

## References

[1] - Chu DM, Ma J, Prince AL, Antony KM, Seferovic MD, Aagaard KM (2017). Maturation of the infant microbiome community structure and function across multiple body sites and in relation to mode of delivery. Nat Med.

[2] - Huurre A, Kalliomäki M, Rautava S, Rinne M, Salminen S, Isolauri E (2008). Mode of delivery – effects on gut microbiota and humoral immunity. Neonatology.

[3] - Sweeney EL, Al-Shehri SS, Cowley DM (2018). The effect of breastmilk and saliva combinations on the vitro growth of oral pathogenic and commensal microorganisms. Sci Rep.

[4] - Dzidic M, Collado MC, Abrahamsson T (2018). Oral microbiome development durin childhood: an ecological succesion influenced by postnatal factors and associated with tooth decay. Iwsme J.

[5] Domininguez-Bello MG, De Jesus-Laboy KM, Shen N (2016). Partial restoration of the microbiota of cesarean-born infants via vaginal microbial transfer. Nat Med.

[6] - Mueller NT, Bakacs E, Combellick J (2015). The infant microbiome development: mom matters. Trends Mol Med.

[7] - Marild K et al. Pregnancy outcome and risk of celiac disease in offspring: a nationwide case-control study Gastroenterology, 142 (2012), pp. 39–45. 10.1053/j.gastro.2011.09.047 PMC3244504 21995948

[8] - Ege MJ (2011). Exposure to environmental microorganisms and childhood asthma N. Engl J Med.

[9] - Algert CS (2009). Perinatal risk factors for early onset of type 1 diabetes in a 2000–2005 birth cohort Diabet. Med.

[10] - Huh SY (2012). Delivery by caesarean section and risk of obesity in preschool age children: a prospective cohort study Arch. Dis Child.

[11] - Mueller NT (2015). Prenatal exposure to antibiotics, cesarean section and risk of childhood obesity. Int J Obes (Lond).

[12] - Metsälä J et al. Prenatal and postnatal exposure to antibiotics and risk of asthma in childhood Clin. Exp. Allergy, 45 (2015), pp. 137–145 The effects of perineal disinfection on infant’s oral microflora after transvaginal examination during delivery BMC Pregnancy Childbirth 2019;19(1):213. 10.1186/s12884-019-2350-3 PMC6591937 31234808

[13] - Collado MC, Rautava S, Aakko J (2016). Human gut colonisation may be iniziated in utero by distinct microbial communities in the placenta and amniotic fluid. Sci Rep.

[14] - Koleva PT, Kim J-S, Scott JA (2015). Microbial programming of health and disease starts during fetal life. Birth Defects Res C Embryo Today.

[15] - Daalderop LA, Wieland BV, Tomsin K (2018). Periodontal disease and pregnancy outcomes: overview of systematic reviews. JDR Clin Trans Res.

[16] - Hill GB (1993). Investigating the source of amniotic fluid isolates of fusobacteria. Clin Infect Dis.

[17] - Puertas A, Magan-Fernandez A, Blanc V (2018). Association of periodontitis with preterm birth and low birth weight: a comprehensive review. J Matern Fetal Neonatal Med.

[18] - Kaan AM, Zaura E (2022). Oral Microbiome Transmission and Infant Feeding Habits. mBio.

[19] - Shaiber A, Willis AD, Delmont TO, Roux S, Chen LX, Schmid AC, Yousef M, Watson AR, Lolans K, Esen ÖC (2020). Functional and genetic markers of niche partitioning among enigmatic members of the human oral microbiome. Genome Biol.

[20] - D’Agostino S, Ferrara E, Valentini G, Stoica SA, Dolci M (2022). Exploring oral microbiome in healthy infants and children: a systematic review. Int J Environ Res Public Health.

[21] Dominguez-Bello MG, Costello EK, Contreras M, Magris M, Hidalgo G, Fierer N, Knight R (2010). Delivery mode shapes the acquisition and structure of the initial microbiota across multiple body habitats in newborns. Proc Natl Acad Sci USA.

[22] - Li F, Fu D, Tao D, Feng X, Wong MCM, Xu W, Lu H (2021). Dynamic Observation of the effect of maternal caries on the oral microbiota of Infants aged 12–24 months. Front Cell Infect Microbiol.

[23] - Tao D, Li F, Feng X, Wong MCM, Lu H (2018). Plaque biofilm microbial diversity in infants aged 12 months and their mothers with or without dental caries: a pilot study. BMC Oral Health.

[24] - Rosemblatt R, Steinberg, Mankuta D, Zini A (2015). Acquired oral microflora of newborns during first 48 hours of life. J Clin Pediatr Dentistry –.

[25] - Hurley E, Mullins D, Barrett MP, O’Shea CA, Kinirons M, Ryan CA, Stanton C, Whelton H, Harris HMB, O’Toole PW (2019). The microbiota of the mother at birth and its influence on the emerging infant oral microbiota from birth to 1 year of age: a cohort study. J Oral Microbiol.

[26] - Li X, Liu Y, Yang X, Li C, Song Z (2022). The oral microbiota: community composition, influencing factors, pathogenesis, and interventions. Front Microbiol.

[27] - Butler CA, Adams GG, Blum J, Byrne SJ, Carpenter L, Gussy MG, Calache H, Catmull DV, Reynolds EC, Dashper SG (2022). Breastmilk influences development and composition of the oral microbiome. J Oral Microbiol.

[28] - Xu Y, Jia YH, Chen L, Huang WM, Yang DQ (2018). Metagenomic analysis of oral microbiome in young children aged 6–8 years living in a rural isolated chinese province. Oral Dis.

[29] – StruzycKa (2014). I the oral microbiome in dental caries. Pol J Microbiol.

[30] - Madianos PN, Bobetsis YA, Offenbacher S (2013). Adverse pregnancy outcomes (APOs) and periodontal disease: pathogenic mechanisms. J Periodontol.

